# Effectiveness of the population-based *Check your health* preventive programme conducted in primary care with 4 years follow-up [the CORE trial]: study protocol for a randomised controlled trial

**DOI:** 10.1186/1745-6215-15-341

**Published:** 2014-08-29

**Authors:** Helle Terkildsen Maindal, Henrik Støvring, Annelli Sandbaek

**Affiliations:** Department of Public Health, Health Promotion and Health Services, Aarhus University, Bartholins Allé 2, 8000 Aarhus C, Denmark; Department of Public Health, Biostatistics, Aarhus University, Nordre Ringgade 1, 8000 Aarhus, Denmark; Department of Public Health, General Practice, Aarhus University, Nordre Ringgade 1, 8000 Aarhus, Denmark

## Abstract

**Background:**

The periodic health check-up has been a fundamental part of routine medical practice for decades, despite a lack of consensus regarding its value in health promotion and disease prevention. A large-scale Danish population-based preventive programme ‘Check your health’ was developed based on available evidence of screening and successive accepted treatment, prevention for diseases and health promotion, and is closely aligned with the current health care system.

The objective of the ‘Check your health’ [CORE] trial is to investigate effectiveness on health outcomes of a preventive health check offered at a population-level to all individuals aged 30–49 years, and to establish the cost-effectiveness.

**Methods/Design:**

The trial will be conducted as a pragmatic household-cluster randomised controlled trial involving 10,505 individuals. All individuals within a well-defined geographical area in the Central Denmark Region, Denmark (DK) were randomised to be offered a preventive health check (Intervention group, n = 5250) or to maintain routine access to healthcare until a delayed intervention (Comparison group, n = 5255). The programme consists of a health examination which yields an individual risk profile, and according to this participants are assigned to one of the following interventions: (a) referral to a health promoting consultation in general practice, (b) behavioural programmes at the local Health Centre, or (c) no need for follow-up.

The primary outcomes at 4 years follow-up are: ten-year-risk of fatal cardiovascular event (Heart-SCORE model), physical activity level (self-report and cardiorespiratory fitness), quality of life (SF12), sick leave and labour market attachment. Cost-effectiveness will be evaluated according to life years gained, direct costs and total health costs. Intention to treat analysis will be performed.

**Discussion:**

Results from the largest Danish health check programme conducted within the current healthcare system, spanning the sectors which share responsibility for the individual, will provide a scientific basis to be used in the development of systems to optimise population health in the 21st century.

**Trial registration:**

The trial has registered at ClinicalTrials.gov with an ID: NCT02028195 (7. March 2014).

## Background

Non-communicable diseases (NCD) such as diabetes and cardiovascular diseases (CVD) are leading causes of reduced life expectancy and quality of life in populations throughout the world. In both industrialised and developing countries the development of NCD is closely linked to prevalent health risk factors for example, low physical activity, obesity and smoking. Furthermore, poor mental health is a growing health concern that affects not only quality of life, but also physical health and work capacity [1]. The periodic health examination has been a fundamental part of medical practice for decades, despite a lack of consensus regarding its value in health promotion and disease prevention [2]. Previous efficacy and modeling studies have indicated that preventive health checks and screening for diabetes and/or CVD are likely to be cost effective [3, 4], and Cooney *et al*. showed how high-risk and population strategies are complementary in the reduction of risk factors for CVD [5]. However, the evidence for health checks covering more conditions jointly and general health as part of routine health care services is inconsistent. The review of Boulware *et al*. [6] suggests that a periodic health check improves delivery of preventive services and may lessen patient worry. A recent Cochrane review [7] found no reduction in morbidity or mortality due to general health checks, neither for CVD or cancer. The review compared a variety of health checks in cancer units, dental care and local communities. However, most trials had considerable methodological problems, and took place 20 to 30 years ago, when the preventive medications in current use had not yet been introduced [8]. Despite the ongoing controversy about the effect of periodical health checks, they are systematically offered on a routine basis in current health care programmes. In the UK the National Health Service Health Check programme has recently been introduced for all citizens aged 40 to 74 years to assess individual risk of heart disease, stroke, kidney disease and diabetes and to support individual risk reduction via individually tailored advice [9, 10]. Similarly, health checks in different variations and settings are conducted in the US/Canada [2, 8]. In 1992, a Danish study, the EBELTOFT health promotion study [11, 12], investigated health checks in general practice in a small area in a population aged 30 to 49 years (approximately 1,500). At the 5-year follow up the effect on cardiovascular risk was reported, and an increase in estimated life expectancy was shown [13]. Wilson and Jungner [14] cautioned that early risk assessment is only of use when accepted treatments for the disease in question are available. Since 1992, knowledge of disease prevention has improved, and a wider range of effective treatment options have become available. Further, the emphasis on a broad health definition encompassing not only disease prevention, but also health promotion in general, so as to ensure physical, mental and social health in general populations, has now become the responsibility of health authorities [15].

The objective of the pragmatic household cluster-randomised controlled trial, Check your health (CORE), is to investigate the effectiveness on health outcomes of preventive health checks offered at a population level to all individuals aged 30 to 49 years in a primary care setting, with shared responsibility between the local municipality and general practice. Furthermore, the objective is to investigate the cost-effectiveness of the preventive health check according to life years gained, direct costs and total health costs.

## Methods

### Study design and population

**Figure 1:**
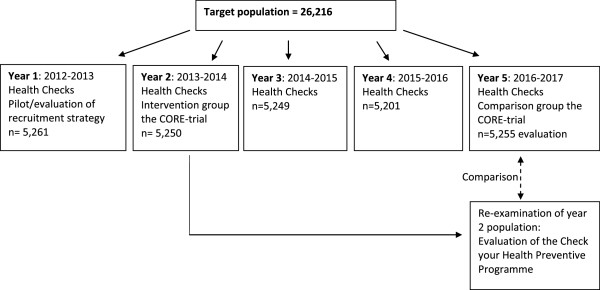
**Participants in the preventive programme, Check your health, Randers, DK 2013.**

The study is designed as a household cluster-randomised pragmatic trial to investigate the effects of preventive health checks offered to all adults aged 30 to 49 years in the municipality of Randers, DK. Administratively, all citizens who were between 30 to 49 years old and living in Randers on January 1, 2012, were identified in the Danish Civil Registers and randomised into five groups (n = 26,216) (Figure 1). The five groups are to be invited to attend the health check in the following five consecutive calendar years. The second (n = 5,250) and the fifth group (n = 5,255) compose the study population for this trial, as the intervention group and the comparison group, respectively. The first group serves as a pilot group for optimising intervention and recruitment procedures, whereas the third and fourth groups are populations to be included in other studies.

The intervention group (I) will be invited for a preventive health check in 2013 to 2014 with follow up after four years in 2016 to 2017, which is the maximal follow-up time within the pragmatic design. At this time the comparison group (C) will be invited completely analogously to the initial invitation of the intervention group. The single-exclusion criterion for not receiving an invitation is terminal illness as reported by the general practitioner (GP). Individuals allocated to the intervention or comparison group, who die before invitation, will be excluded.

### Randomisation

Randomisation was performed on clusters defined by households based on addresses obtained from the Danish Civil Register. Cluster randomisation by household was chosen to minimise the expected contamination between participants living together, because motivation to change behaviour after the health checks would potentially impact the entire household. The randomisation was further balanced on GP level so as to even out the practice workload over the scheduled five years of the programme. Of 26,216 individuals living in the municipality of Randers, 913 (3.5%) were not affiliated with a GP within the municipality of Randers. Consequently, these were allocated to an imaginary GP in the randomisation procedure. For each of the 17,881 households a random index individual was chosen and then randomised to receive an invitation to a health check in one of the scheduled five calendar years. All cohabitants were randomised to the same group regardless of their GP affiliation. Thus, all individuals within the same cluster were invited to a health check at the same point in time. Randomisation was done by an independent statistician. Recruitment for the CORE trial started in summer 2013.

### Setting

In Denmark the local and regional health authorities share responsibility for disease prevention. The preventive health checks are conducted in Randers municipality in collaboration between the local Randers Health Centre (RHC) and the GPs, with the GPs signing the invitation. The health examinations are undertaken at RHC in a test clinic established for the Check your health preventive programme. The RHC has an open-door policy for all citizens in the geographical area. The centre offers a variety of health services, including disease management programmes, smoking cessation, dental services and pharmacy. In DK, general practice is the primary entry point into the health care system, and this tax-funded health care system ensures free access for all citizens to general practice services. Danish GPs operate as independent contractors within the public health service and are remunerated based on a combination of fee-for-service (2/3) and capitation basis (1/3) [16].

### Intervention

#### Comparison group: standard prevention and treatment strategy followed by lagged intervention

The comparison group is offered standard treatment with free access to the Danish healthcare system and the open-hour preventive services at the local RHC until the end of the study period (2017 to 2018), in which the intervention will be offered. The health examination of the intervention will provide data for the outcome measurements in the randomised trial.

#### Intervention group: health examination and risk-stratified follow-up intervention

**Figure 2:**
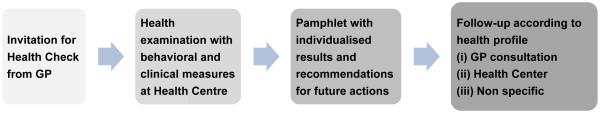
**The four components of the preventive programme, Check your health, Randers, Denmark 2013.** GP, general practitioner.

The Check your health programme was designed to promote awareness of and action on health behaviour and health condition. The programme includes four components: (I) an invitation; (II) a health examination including clinical measures and questionnaires; (III) an individualised health profile pamphlet; and (IV) an offer of further intervention depending on the risk-profile. The follow-up intervention is flexible with different options stratified by the risk profile of the individual (Figure 2).

### Invitation

All individuals will receive a postal invitation including information about the objectives of the Check your health preventive programme and the content of the health examination. A suggested appointment time is provided. The scheduled time may be accepted, modified or rejected via phone or Internet. If the appointment is not accepted within 7 days, a reminder is sent out with a new appointment time. A second reminder is sent 3 weeks after the first reminder.

### Health examination

#### Questionnaire

Prior to the health examination participants are asked to answer a web-based questionnaire, which may be completed in approximately 20 minutes. The questions concern self-reported health (short form 12 (SF12)) and mental health (SF12 mcs) [17], physical activity level, smoking habits and alcohol use, re-using items from the Danish National Health Profile questionnaire [18] including alcohol risk-behaviour (AUDIT) [19] (Table 1). Participants are informed about the opportunity for responding to the questionnaire at the RHC if support is needed for any reason.

**Table 1:** **Measurements in the health examination in the preventive programme, Check your health, including technical information and levels for follow up at the local Health Centre (RHC) or their general practitioner (GP)**

	Measurement	Technical information	Levels for referral
**Clinical measures**	BP (mmHg) - systolic and diastolic	Measured in sitting position at left arm, three times.	To GP:
		Average of three measurements	If systolic BP ≥140 or
		Device: Omron M6 blood pressure monitor, Omron Healthcare Europe BV	Diastolic BP ≥95
			Immediate referral: 200/120
	Height (cm), weight (kg)	Device: Seca 222 mechanical telescopic measuring rod, Seca, Germany	
	BMI (kg/m^2^)		To RHC:
			If BMI ≥28 kg/m^2^
	Waist (cm)	Device: Seca 203 measuring band, Seca, Deutschland	
		Quality assurance: inter/intra validation, yearly	
	Lung function/spirometry	Device: EasyOne Diagnostic Spirometer, NDD Medical Tecnologies, inc. MA, USA	To GP:
	FVC (L,%), FEV_1_ (L,%), FEV/_1_FVC		If FEV_1_ or FVC ≤80% or
		Quality assurance: daily calibration, yearly intra/intervariation measurements	FEV_1_/FVC ≤0.70
	Physical fitness (ml O_2_/kg/min)	Aastrand 1 point submaximal test (ref 20)	To RHC:
			For men age 30 to 39 yrs: <35
			For men age 40 to 49 yrs: <31
		Device: Monark 939 E Pendulum Ergometer, Monark Exercise AB, Sweden	For women age 30 to 39 yrs: <28
		Quality assurance: daily calibration. Yearly external calibration check. Yearly intra/intervariation measurements	For women age 40 to 49 years: <26
**Patient reported by questionnaire**	Smoking	Questionnaire: daily, weekly, less than weekly, quitted, never	To RHC:
			If current smoker
	Alcohol use	AUDIT measures risk behaviour. (ref 19)	To GP:
			For men if audit score ≥8 or
			NAU ≥21 per week
			For women if audit score ≥8* or
			NAU ≥14 per week
		Number of alchohol units (NAU) per week is estimated on basis of reported number per weekday.	*During the first 6 months the referring score was ≥6.
	Mental Health	SF-12 mcs 2007. Lincoln, RI: QualityMetric Incorporated, 2007.(ref 17)	To GP:
			If mental component score <35.76
	Self-reported general health	Question 1 from SF-12:	To GP:
		In general, would you say your health is:	If answer is "Fair" or "Poor"
		Excellent, Very good, Good, Fair, Poor	
**Biochemical**	HbA1c	Measured in capillary blood	To GP:
		Device: DCA Vantage Analyzer, Siemens Healthcare, Siemens AG, Germany	If HbA1c ≥6,0%/42 mmol/mol
		Quality assurance: monthly control test. Every three months: optical calibration test according to manufacturer guidelines.	Immediate referral: HbA1c ≥8,0%/63 mmol/mol
	Lipid profile (total cholesterol, HDL, LDL, triglyceride)	Measured in capillary blood	To GP:
		Device: Alere Cholestech LDX System, Alere Denmark	If total cholesterol > =6 mmol/l or
		Quality assurance: Daily calibration. Yearly calibration check according to manufacturer guidelines	LDL ≥6 mmol/l
**Aggregated measure**	Risk of CVD within 10 years	Measured by SCORE (ref 21), includes information on systolic blood pressure, Total cholesterol, smoking, gender and age. Denmark is categorized as a low-risk country.	To GP:
			If score ≥5%

BP, blood pressure; BMI, body mass index; FVC, forced vital capacity; FEV_1_, forced expiratory volume at one second; HbA1c, glycated haemoglobin; HDL, high-density lipoprotein; LDL, low-density lipoprotein; CVD, cardiovascular disease; AUDIT, alcohol use disorders identification test; WHO, World Health Organisation; NAU, number of alcohol units; SF12 mcs, Short form 12 mental component score.

#### Clinical examination

The health professionals at RHC administer the clinical examination. Participants may wear everyday clothing for the examination, which takes approximately 40 minutes. The following measures will be taken: biochemical (glycated haemoglobin (HbA1c), total-cholesterol, low-density lipoprotein (LDL)), systolic and diastolic blood pressure (SBP and DBP), spirometer (lung function test) and Aastrands submaximal bike-test (cardiorespiratory fitness) [20]. All biochemical measures are analysed on location with the following measurements: DCA Vantage Analyzer, Siemens Healthcare, Siemens AG, Germany (HbA1c), and Alere Cholestech LDX System, Alere Denmark (lipids). A CVD risk score is calculated [21, 22]. Details about the measurements are given in Table 1.

### An individualised health profile pamphlet

Immediately after the health examination participants receive their printed individual health profile pamphlet with the results of each measurement, the CVD risk score, and an overall assessment of their health condition. Furthermore, recommendations for follow up according to their health profile are given (Table 1). Data from the health examination, including the questionnaire responses, are transferred directly into the patient’s file at their GP's office. GPs are contacted immediately if SBP is above 200 mmHg, if DBP is above 120 mmHg or if HbA1c is above 8% (63 mmol/mol).

### Follow up according to risk profile

Stratification based on the individual health profile determines whether the follow-up intervention for the participant should be either (a) referral to a health-promoting consultation in general practice, (b) targeted behavioural programmes at RHC, or (c) no identified need for health-promoting follow up. The algorithm for the stratification is outlined in Table 1. If referred to general practice, participants will have to make an appointment themselves in line with standard procedures for general practice in DK. The health-promoting consultation is based on shared decision-making based on a shared agenda [23]. National clinical guidelines govern how the patients are to be treated in general practice. The GP has the opportunity to refer to chronic disease programmes or behavioural programmes at RHC targeted at participants in the Check your health programme. For each consultation the GP is paid 50 Euros based on a specific and tailored economic remuneration agreement between the Health Authorities and the General Practice Association (§2 agreement). Follow-up consultation in the case of, for example, hypertension is paid according to general agreements. All consultations are monitored by regional data for the use of general practice services. The behavioural interventions tailored to the participants are provided in the local health centre RHC and are: healthy diet (10 meetings of 1½ hours over months); smoking cessation (one to two individual counselling sessions in person or by phone or mail); a motivational interview about physical activity (1½ hours with follow up by phone or mail); alcohol abuse (1½ hours with follow up by phone or mail); mental health (1½ hours individual counselling and 7 group meetings of 2½ hours). A number of disease-specific self-management courses are offered to all citizens with chronic obstructive pulmonary disease, diabetes, CVD or musculoskeletal conditions.

### Health professional training

Before enrolment of patients into the CORE trial, all GPs and health professionals at RHC received information on the trial and how the full programme was to be delivered, and they were offered formal training according to their tasks in the programme. The GPs and their staff were invited to participate in four after-work meetings of 2 hours each for a total of 8 hours before the invitation of the intervention group. The agenda of the four meetings were: introduction to the programme, the concept of shared decision-making, how to promote physical activity, and establishing consensus on the risk stratification used for follow up. During the study period the GPs will receive newsletters (mail and email) and an invitation to visit a dedicated website with information on all procedures and news in the study. The total education offered to each GP was approximately 10 hours for the trial, combined with the options of receiving advice and follow up upon individual request.

The health professionals at RHC were trained in all measurement procedures, as well as health promotion and risk communication before the start of the programme. The training was partly defined to ensure standardisation and quality, and partly tailored to fit individual competencies and requests due to different educational backgrounds, for example, Master of Sports Science, Master of Public Health, nurse, and physiotherapist. Furthermore, the staff participated in meetings with the research team to ensure the correct handling of data (two meetings of 2 hours each). In the intervention period the staff will receive education on evidence and feedback for handling of risk conditions such as CVD, hyperglycaemia and diabetes, sedentary lifestyle and low fitness, alcohol risk behaviour, poor mental health, obesity and insufficient lung function. The meetings will be provided every second month. The total education consists of approximately 50 hours.

### Collaboration and involvement of stakeholders

A steering committee was established with members from The Central Denmark Region, health department, the municipality responsible for the health centre, the GPs and the research team, with the aim of developing the programme, ensuring implementation and monitoring the budget.

### Outcomes

The outcomes of the CORE trial reflect different aspects of a broad health concept (cardiovascular risk, physical activity level and self-reported health) and functional capacity (affiliation to the labour market). Cardiovascular risk will be measured as the individual ten-year-risk of a fatal cardiovascular event as estimated by the European Heart-SCORE from age, gender, smoking status, SBP and total cholesterol [21]. Physical activity level will be measured by (1) self-reported physical activity (days/week with minimum 30 minutes moderate physical activity) and (2) cardiorespiratory fitness, measured by Aastrands submaximal bike-test [20]. Health-related quality of life will be measured by the medical outcome study short form 12 health survey (SF12) [24].

Functional capacity will be measured as affiliation to the labour market (work participation in the last year) [25], taking into account whether non-affiliation is self-selected. Information on working status will be collected from a national register of social transfer payments (DREAM), which includes information on all public transfer payments administered by Danish ministries and municipalities for Danish citizens on a weekly basis since 1991. The type of transfer payment is recorded for each week in which the individual received a benefit for at least 1 day. At present the DREAM database includes 114 different codes for social transfer payments. If no transfer income is recorded for a specific week, the individual is considered to be self-supporting or on short-term sick leave (less than 3 weeks). According to sick leave a citizen in the workforce in DK (employed as well as unemployed) is entitled to sickness absence compensation (at the time of this study after 3 weeks of illness), and in case the employee receives normal salary during sick leave, the employer receives municipal reimbursement. Number of sick days last year was obtained from the Danish Integrated Database for Labour Market Research (IDA). In DK each person has a unique civil registration number, which allows unique linkage across all registers. It is thus possible to get information on each person according to not only sociodemographic data and the above mentioned social transfer data, but also regarding diagnoses obtained from inpatient contacts (ICD10 codes) and medication use from the Register of Medicinal Product Statistics. Researchers will be blinded to the civil registration number, as data are linked at Denmark Statistics.

### The cost-effectiveness evaluation

The economic benefits of the interventions will be examined in terms of life-years saved and costs. Cost-effectiveness and efficiency will be determined by comparing mean direct and total costs (direct and productivity costs) and expected life years gained (LYG) in the intervention group with the comparison group. Direct costs are intervention and implementation costs, the costs of medicine and somatic and psychiatric inpatient and outpatient hospital services as well as services from practitioners (that is, GPs, specialists, dentists, physiotherapists, chiropractors, opticians, et cetera). The productivity costs include morbidity costs (days absent from work, rehabilitation, and early retirement) and mortality costs, stratified by 5-year age groups and gender. Cost data will be obtained from Danish registers and include socioeconomic factors such as education, income, marital/cohabitation status, and ethnicity.

### Data obtainment

Measurements at 4-year follow-up (biochemical, anthropometric and questionnaire) will be undertaken by trained staff unaware of study group allocation, and following standard operating procedures, as in the detailed description of the health examination. Historic register data detailing sociodemographic information, prescriptions, contacts to GPs, diagnosis from hospital admissions and workforce will be obtained via Statistics Denmark. Furthermore, similar data will be obtained for a corresponding population in a comparable municipality to allow control for the impact of time trends in relevant health outcomes and externally imposed changes in preventive services and other procedures.

### Sample size

The sample size was determined as the number of people that could be examined in the municipality per year. With 10,505 enrolled in the trial, equally distributed between the two groups, an expected standard error (SE) of their average difference in days per week with minimum 30 minutes moderate physical activity was found to be 0.19 days per week. For SBP the expected SE is 1.30 mmHg. The SEs take into account an expected loss to follow up of 30%, as well as clustering due to individuals being allocated to one of approximately 40 different participating GP units (based on an assumed intracluster correlation coefficient (ICC) of 0.01, the design effect due to clustering is nearly four) [26]. Estimates of the variability between individuals in physical activity and SBP were obtained from recent population-based Danish studies [18, 27]. For physical activity, the analysis will use robust variance estimates to account for the non-normally distributed outcome, while the analysis of SBP will be adjusted for age. If the observed ICC exceeds 0.01, study precision will benefit from making comparisons within GP units, and the above SEs can therefore be considered conservative. The power to detect a difference of half a day extra per week with a minimum of 30 minutes moderate physical activity exceeds 90% (α = 0.05).

### Statistical methods for health outcomes

The effects will be analysed according to the intention-to-treat principle with allowance for household clusters and with the following outcomes examined at the time of follow up (re-examination) for both groups: sick leave will be measured as a dichotomous variable, and as categories of number of sick-leave periods over 3 weeks. This analysis will be adjusted for having received other government-paid pensions, such as early retirement. Work participation will be described as a fraction of full-time employment in the 52 weeks before end of follow up. Adjustments, for example, for education and maternal leave, will be made.

### Statistical method for the health economic evaluation

The cost-effectiveness analysis is based on individual-level data, and the analyses are based on the intention-to-treat principle with allowance for clustering as above. The health economic analyses will compare the LYG relative to the mean direct and total costs (direct and productivity costs) between the intervention group and the control group over a four year period. For different social groups the clinical effect, LYG, the healthcare utilisation, and the direct costs and productivity costs per participant, respectively, will be estimated and compared. Additionally, the annual intervention costs will be estimated for each of the first 3 years for the purpose of giving the municipality and the region an overview of their expenses. The analyses will be reported with significance and confidence intervals based on generalized linear models or bootstrapping. Cost effectiveness acceptability curves are plotted for a given threshold.

### Legal and ethical aspects

The preventive programme, Check your health, is considered part of the routine activities running in RHC. The CORE trial has been presented to The Scientific Ethics Committee, Central Denmark and according to law number 593 of 14 June 2011, which relates to the scientific ethical treatment of health scientific research in DK; the used data are considered routine data and thus, ethical approval is not needed when used for the trial. The study will comply with the Helsinki Declaration. In agreement with the Danish Health Law each participant prior to their health examination has provided written informed consent for data to be used for research purposes., and written informed consent to use data for research properties will be obtained from all individuals, according to the Danish Health Law. The Danish Data Protection Agency has approved the storage of data at the Department of Public Health at Aarhus University (reference number 2012-41-0183). The trial protocol has been registered at ClincalTrials.gov (identifier NCT02028195).

## Discussion

The large-scale Danish population-based preventive health-check programme, Check your health, targeting all citizens aged 30 to 49 years in a well-defined area, has been developed. The programme is based on available evidence on screening and subsequently accepted treatment, prevention of diseases and health promotion closely aligned with the current health care system. The pragmatic trial has been designed to evaluate a complex primary-care intervention, which contains invitation, examination/questionnaire, individualised health profile and follow up. Pragmatic trials [28] answer questions about effectiveness by determining the effect on current healthcare, and are in that respect directly relevant to participants, funders, communities, and healthcare professionals. The availability of a well-defined population in a well-defined area is a major strength in this study as the entire population - not only participants - can be followed in registers. This allows direct study of the external generalizability and offers the possibility of conducting intention-to-treat analyses on selected outcomes with virtually no loss to follow up.

### Choice of evaluation and effect measures

This study will evaluate the effect of health checks in a general population in a 4-year follow-up period. The logistics of the trial would not in any case allow the intervention to be offered simultaneously to the entire target population. The stepped inclusion in the trial (lagged intervention) is, therefore, considered to be the strongest design to evaluate a realistic health-promotion strategy within a well-defined geographical area - the municipality. The trial will not solely assess effectiveness of the intervention with respect to risk factors, but effectiveness of a broad range of health and social outcomes, and the trial establishes the associated costs. While preventive services can be costly in the short-term, they are intended to lower overall healthcare expenditures over time by addressing potential health problems sooner rather than later. A recent European model study showed that a health check assessing diabetes, hypertension, lipids and smoking would be cost effective in the six countries included, Denmark, France, Italy, Germany, Poland, and the UK [3]. As part of the CORE trial a process evaluation will be conducted, including awareness of the ethical and legal issues that might arise from disease- and risk-screening as part of the health check, as well as from the health interview. Any screening programme has the potential to label an asymptomatic person as a patient. Being labelled with a risk factor or a disease could cause anxiety and have adverse effects. Studies related to screening of individuals for chronic diseases and conditions have shown that the level of psychological stress induced by screening is limited and short lasting [29].

### Precision

As the CORE trial involves more than 5,000 participants in each of the two groups, high precision can be expected for effect estimates with respect to each of the outcomes of interest. While the primary outcome of time spent on exercise is self-reported, and thus may be prone to recall bias, the use of objectively measured outcomes and inclusion of register data will allow comparisons without this potential misclassification.

### Feasibility

As the study strategy is chosen to be pragmatic from the outset, the study is feasible. Sub-studies will, however, investigate non-participation, which may threaten overall generalizability, as well as relevance to certain specific sub-groups determined by, for example, age, social class and initial health. Non-participation in preventive healthcare is a well-known challenge [30]. In this study the participants are invited by their GP to the health check, which in former studies has been shown to stimulate a high level of uptake [31]. However, characterising non-participants is crucial in evaluating any population-based intervention. Besides obtaining register data for all non-participants and participants, this study will explore the uptake, views and experiences of invited individuals with qualitative methods and descriptive statistics at the three levels of the individual, health professional and organisational and leadership practice. A considerable diversity in implementation of the UK National Health Services Health Check has been reported among general practices [32]. This study overcomes this by being locally anchored with a formal quality assurance process in place in order to optimise the implementation of the programme. The implementation will take into account the international literature on the organisation of health services, which emphasizes that the success of initiatives such as health checks tend to build on collaboration among a variety of organisations/professionals, as well as well-functioning inter-organisational relations [33].

### Limitations of study design

As with most community-based interventions this study is at risk of having its effect diluted by contamination [34]. At the individual level, contamination may occur when participants allocated to the comparison group opt for a health check prior to their designated appointment or adopt changes in lifestyle induced by their peers in the intervention group. Household randomisation may reduce the extent of contamination. However, participating in social groups and community activities as such may still lead to contamination. In the study period, GPs will increase their awareness of health checks. This may lead to either postponing health checks for some patients or inviting some patient earlier than designated with an increase in requests for health checks as the consequence. Although GPs will be asked not to change their practice, it would be regarded unethical to deny patients and/or GPs this option. The magnitude of this possible contamination effect will be analysed using data on consultations obtained from the National Health Service Register. Furthermore, contamination can also occur by the extra attention paid by Randers Municipality to physical activity initiatives and other health promotion activities over the study period. This will be addressed by using the unique registers in Denmark to compare the health status in Randers municipality with similar municipalities matched by age, gender, and social-demography. Similarly, secular trends in health attitude and behaviour over the 4-year follow-up period may affect health status in the control group before their first health check, which is the measurement used for comparison with the intervention group. These trends may dilute or inflate the apparent effect if they are not mirrored by similar changes in the intervention group. Differences in health trends between the intervention and comparison group can, however, to some extent be explored and controlled for by our use of national routine register data over the study period.

### Interpretation of results

Results from the largest Danish health-check preventive programme conducted within the current healthcare system, spanning the sectors that share responsibility for the individual, will provide a scientific basis that is of immediate relevance for primary care health services priorities. The anticipated high validity of the CORE trial will provide evidence about the benefits, uptake, harm and costs of preventive health checks studied over a 4-year period. Further the trial design allows study of whether preventive health checks provide sustained effects on disease patterns and mortality. Such evidence is required when attempting to develop health care systems that may optimise population health in the 21st century.

## Trial status

The trial at the time of submission of this manuscript has enrolled approximately 2,000 participants. Recruitment is ongoing.

## Abbreviations

AUDIT: alcohol use disorders identification test
BMI: body mass index
CORE-trial: Check your health trial
CVD: cardiovascular disease
DBP: diastolic blood pressure
DK: Denmark
DREAM: national register of social transfer payments
FEV_1_: forced expiratory volume at one second
FVC: forced vital capacity
GP: general practitioner
HbA1c: glycated haemoglobin
HDL: high-density lipoprotein
ICC: intercluster coefficient
LDL: low-density lipoprotein
LYG: expected life years gained
METS: metabolic equivalent of tasks
NCD: non-communicable diseases
RHC: Randers Health Centre
SBP: systolic blood pressure
SE: standard error
SF12: health status short form 12 version
pcs: physical component score
mcs: mental component score
WHO: World Health Organisation.
